# Sex differences involved in persistent atrial fibrillation recurrence after radiofrequency ablation

**DOI:** 10.1186/s12872-022-03002-z

**Published:** 2022-12-16

**Authors:** Haiwei Li, Zefeng Wang, Zichao Cheng, Yingming Zhu, Zhongyu Yuan, Jianwei Gao, Xiaoping Zhang, Yongquan Wu

**Affiliations:** 1 Beijing Anzhen Hospital, Capital Medical University, Beijing, People’s Republic of China; 2 Department of Cardiology, Beijing Anzhen Hospital, Capital Medical University, No. 2 Anzhen Road, Chaoyang District, Beijing, 100029 People’s Republic of China; 3 Department of Radiation Oncology, National Cancer Center/National Clinical Research Center for Cancer/Cancer Hospital, Chinese Academy of Medical Sciences and Peking Union Medical College, Beijing, People’s Republic of China; 4 Beijing Institute of Heart, Lung & Blood Vessel Disease, No. 2 Anzhen Road, Chaoyang District, Beijing, 100029 People’s Republic of China; 5 The Key Laboratory of Remodeling-Related Cardiovascular Diseases, Ministry of Education, Beijing, People’s Republic of China

**Keywords:** Sex differences, Persistent atrial fibrillation, Radiofrequency ablation, Recurrence

## Abstract

**Background:**

In recent years, the difference in outcomes of radiofrequency catheter ablation (RFCA) in persistent atrial fibrillation patients has risen. In particular, biological sex seems involved in a different response to the AF ablation procedure. In our study, we analyzed the AF recurrences after RFCA assessing the other association between male/female patients with the outcomes.

**Methods:**

We enrolled 106 patients (74.5% men) with persistent atrial fibrillation with scheduled follow-up. The baseline clinical characteristics and AF recurrence after RFCA were compared between men and women. Cox regression analyses were performed to determine the risk predictors of AF recurrence.

**Results:**

The proportion of RFCA in women was lower than that in men. Men with persistent AF were younger than women (58.6 ± 10.4 years vs. 65.1 ± 8.7 years, respectively; *p* = 0.003). The left atrium (LA) diameter was higher in males (43.7 ± 4.6 mm vs. 41.3 ± 5.5 mm; *p* = 0.028), and the level of left heart ejection fraction (LVEF) was higher in females (59.4 ± 6.9% vs. 64.1 ± 5.5%; *p* = 0.001). Sex differences in AF recurrence after RFCA were significant during the median 24.4-month (interquartile range: 15.2–30.6 months) follow-up period, and the recurrence rate of AF in women was significantly higher than that in men (*p* = 0.005). Univariable Cox regression analysis showed that female sex was a risk factor for persistent AF recurrence after RFCA [HR: 2.099 (1.087–4.053)]. Univariate Cox regression analysis revealed that non-PV ablation not associated with AF recurrence [HR: 1.003 (0.516–1.947)].

**Conclusion:**

In a monocentric cohort of persistent AF patients, the female biological sex was associated with a higher risk of AF recurrence after RFCA.

**Supplementary Information:**

The online version contains supplementary material available at 10.1186/s12872-022-03002-z.

## Background

Atrial fibrillation (AF) is the most common sustained cardiac arrhythmia worldwide. Recent studies have shown that the estimated prevalence of AF in adults is between 2 and 4%. AF occurrence associated with variety risk factors including demographic factors, health factors and other clinical diseases [1–3]. Similar to other cardiovascular diseases, sex differences exist in patients with AF. Previous studies have demonstrated that the incidence of AF is higher in men than in women. The onset age of AF in men is higher than that in women, but women with AF have a greater symptom burden [4]. Additionally, women undergo ablation less frequently than men, although they typically have a greater symptom burden [5]. Studies of AF catheter ablation have also shown significant sex-based differences, including a higher risk of AF recurrence rate and increased periprocedural complications and hospitalization in women compared with men [5, 6].

Despite being extensively researched, sex-based differences remain poorly understood. Until now, whether these differences are due to sex-based pathophysiological differences, differences in clinical characteristics, or unreasonable bias has been unclear. Additionally, AF management and long-term outcomes seem to be significant across countries.

In the current study, we evaluated sex-based differences in persistent AF, focusing on comorbidities, clinical presentation, and laboratory examination. We also sought to identify predictors of the long-term recurrence of AF.

## Methods

### Design and sample selection

Between January 2019 and December 2020, consecutive patients aged ≥ 18 years who underwent initial RFCA in the Department of Cardiology of Beijing AnZhen Hospital were reviewed. The inclusion criteria were as follows: (1) patients > 18 years of age; (2) patients were diagnosed persistent AF and underwent first RFCA treatment; (3) signed informed consent at admission. The exclusion criteria were: (1) presence of other cardiovascular disease, including abnormal cardiac structures disease, heart failure, and history of CABG (coronary artery bypass grafting), RFCA(radiofrequency catheter ablation), and other cardiac surgical; (2) Incomplete medical history or follow-up data; (3) Other disease including mental disease, advanced malignant tumor, acute, severe renal dysfunction and chronic inflammatory diseases, and autoimmune diseases; (4) Pregnancy; (5) LA anteroposterior diameter > 50 mm. The study was designed and performed in accordance with the Declaration of Helsinki for Human Research and was approved by the Beijing Anzhen Hospital Ethics Committee (Approval NO: 2022042X).

### Data collection

Clinical and laboratory data of all patients were collected, which are including: (1) general clinical data: age, gender, body mass index (BMI, kg/m^2^), comorbidities and calculation of EHRA score, CHA2DS2-VASc score, HAS-BLED score, history of medication and echocardiographic parameters (Echocardiography were performed with a Philips 7C color Doppler ultrasound); (2) Laboratory examinations (results of fasting blood sample obtained on the latest preoperative morning): white blood cell, red blood cell, platelet count, hemoglobin, estimated Glomerular filtration rate, fasting plasma glucose, homocysteine, uric acid, B-type natriuretic peptide, alanine aminotransferase, aspartate transaminase, lactate dehydrogenase, albumin, total bilirubin, total cholesterol, low-density lipoprotein cholesterol.

### Radiofrequency catheter ablation (RFCA) procedure

All patient underwent anticoagulation administration and transesophageal echocardiography (TEE) to exclude atrial thrombus before RFCA. Catheter ablation procedures were performed after obtain patient agreement form to treatment signing. CPVI (circumferential pulmonary vein isolation) were performed for all participants and selective additional atrial ablation (ie, non-pulmonary veins foci ablation) was only performed in patients with AF persisting despite completion of CPVI. Point-by-point CPVI was performed using irrigated ablation catheters in a power control mode at 35 W (irrigation flow 17 mL/min). The ablation index (AI) had a target value of 500 for the anterior LA wall and 400 for the posterior wall. Intravenous heparin was administered continuously to maintain an activated clotting time between 250 and 350 s after transseptal puncture. an irrigation catheter was used in combination with a 3-dimensional mapping system (CARTO, Biosense Webster) conducted using PentaRay. The electrophysiological end point of CPVI was a bidirectional conduction block between the left atrium (LA) and PVs.

### Follow-up

Antiarrhythmic and anticoagulant treatments were maintained for at least 3 months after RFCA. Periodic follow-up visits were conducted at 3, 6, 12, 24 and 36 months in outpatient clinics. Each visit includes medical and physical examination, 12-lead electrocardiogram (ECG) and 24 h Holter monitoring. The endpoint was AF recurrence defined as any documented atrial tachyarrhythmia (atrial fibrillation, atrial flutter, or atrial tachycardia) episode lasting for at least 30 s after RFCA, excluding 3-month blanking period. Follow-up records were based on telephone interviews, and patients' preiodic visits to outpatient clinics at the Beijing Anzhen Hospital and outcomes were judged by trained study personnel and cardiologists. They were strongly recommended to visit the closest hospital for an ECG if they felt discomfort due to cardiac arrhythmia.

### Statistical analysis

Participants were divided into 2 groups according to gender. Continuous variables with normal distribution are expressed as mean ± standard deviation (SD), and non-normally distributed variables were described as median and interquartile range (IQR). Comparisons of means between groups were analyzed using the independent sample t-test for normally distributed data and the Mann–Whitney test for non-normally distributed data. Categorical data were presented as frequencies or percentages and compared between groups using the chi-squared test or Fisher’s exact test. Univariate Cox regression analyses were performed to determine risk factors for persistent AF recurrence, and the hazard ratio (HR) and 95% CI were calculated. Adjusted model were done based age, comorbidities, BMI, LVEF, EHRA score, CHA2DS2-VASc score, and HAS-BLED score to exclude confounding factors. Time-dependent survival between groups (male and female) was evaluated using Kaplan–Meier curves and the Log-rank test. All data were analyzed with SPSS 20.0 (IBM Corp., Armonk, NY, USA), R (version 4.0.4). Two-tailed values of *p* < 0.05 were considered statistically significant.

## Results

### Baseline clinical characteristics

**Fig. 1:**
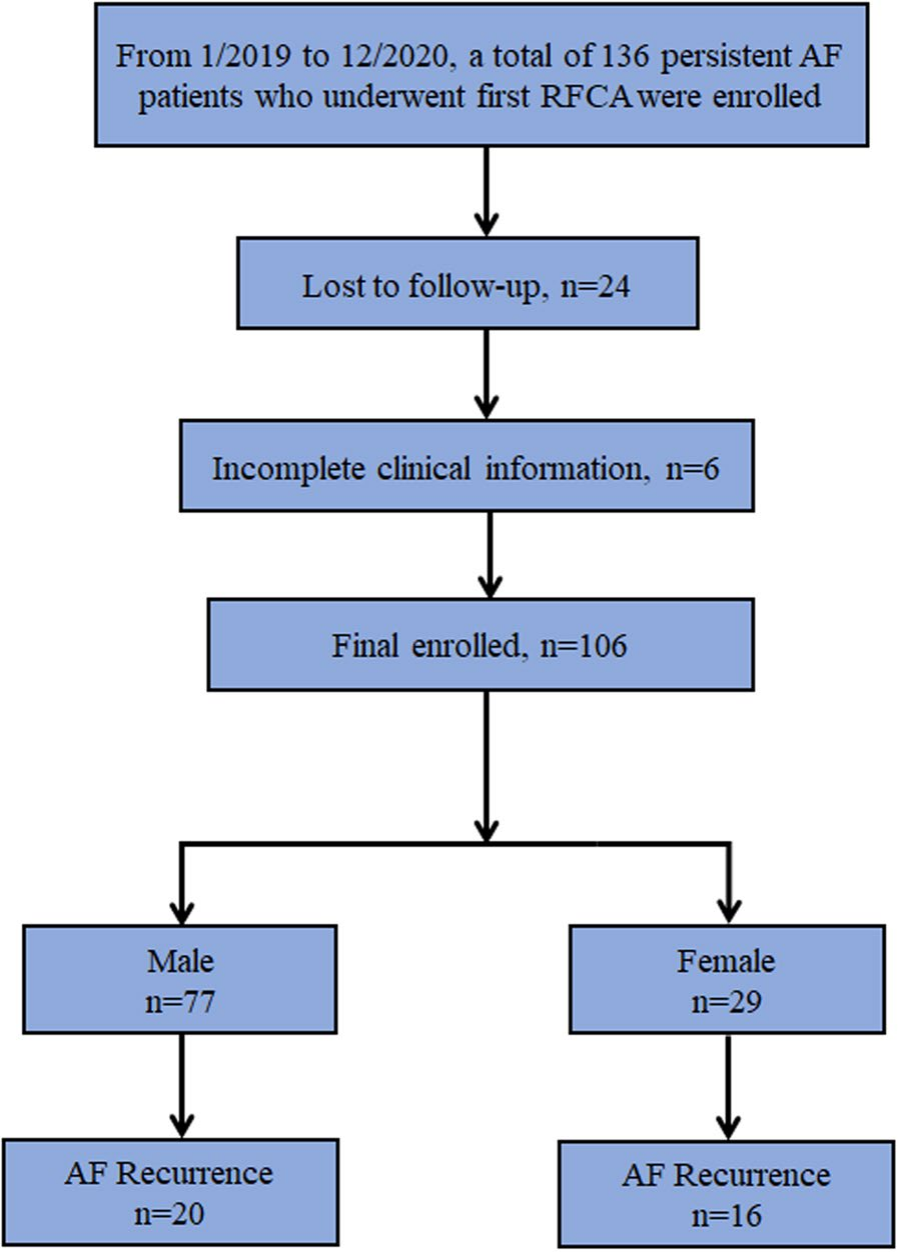
Flow diagram showing population selection and study design

A total of 136 patients were included in this study; 24 patients were lost to follow-up, and 6 patients with incomplete clinical information were excluded (Fig. 1). As shown in Table 1, 106 consecutive persistent AF patients (aged 60.4 ± 10.3 years) were recruited, 77 men (aged 58.6 ± 10.4 years) and 29 women (aged 65.1 ± 8.7 years). The proportion of men undergoing RFCA was higher than that of women, and the age of female patients with RFCA was significantly higher than that of male patients (*p* = 0.003). The proportion of women with CHA2DS2-VASc scores ≥ 2 was significantly higher than that of men (Table 1) (*p* < 0.05). The LA diameter was higher in men (43.7 ± 4.6 mm vs. 41.3 ± 5.5 mm; *p* = 0.028), and the level of LVEF was higher in women (59.4 ± 6.9% vs. 64.1 ± 5.5%; *p* = 0.001). Significant sex differences in AF recurrence after RFCA procedures were found, and the recurrence rate of persistent AF in women was significantly higher than that in men (*p* = 0.005). The levels of RBC and Hb in the male group were significantly higher than those in the female group (Additional file 2: Table S2) (*p* < 0.05). No significant differences were found in other clinical characteristics, including the body mass index (BMI), medical history, the EHRA score, the HAS-BLED score, and laboratory examination, between the male and female groups (all *p* > 0.05).

**Table 1:** Baseline clinical characteristics of the persistent AF stratified according to gender

Characteristic	Total (106)	Male (77)	Female (29)	*p* value
Age, years	60.4 ± 10.3	58.6 ± 10.4	65.1 ± 8.7	0.003
BMI, kg/m^2^	27.1 ± 3.9	27.3 ± 4.0	26.6 ± 3.8	0.398
AF duration, months	43.0 ± 46.6	43.6 ± 48.6	41.4 ± 41.5	0.831
*Medical history*				
CAD, n (%)	12 (11.3)	10 (13)	2 (6.9)	0.38
HTN, n (%)	64 (60.4)	49 (63.6)	15 (51.7)	0.266
DM, n (%)	15 (14.2)	13 (16.9)	2 (6.9)	0.191
stroke, n (%)	11 (10.4)	6 (7.8)	5 (17.2)	0.157
*Medical therapy at admission*				
Statins, n (%)	24 (22.6)	20 (26)	4 (13.8)	0.184
ACEI/ARB, n (%)	23 (21.7)	15 (19.5)	8 (27.6)	0.369
CCB, n (%)	21 (19.8)	14 (18.2)	7 (24.1)	0.495
Beta-block, n (%)	22 (19.8)	17 (22.1)	5 (17.2)	0.586
*AF related score*				
EHRA score				0.886
1, n (%)	12 (11.3)	8 (10.4)	4 (13.8)	
2, n (%)	58 (54.7)	43 (55.8)	15 (51.7)	
3, n (%)	36 (34)	26 (33.8)	10 (34.5)	
*CHA2DS2-VASc score*				< 0.001
0 or 1, n (%)	57 (53.8)	50 (64.9)	11 (37.9)	
≥ 2, n (%)	49 (46.2)	27 (35.1)	18 (62.1)	
HAS-BLED score ≥ 3, n (%)	4 (3.8)	3 (3.9)	1 (3.4)	0.915
*Echocardiographic parameters*				
LAd, mm	43.0 ± 5.0	43.7 ± 4.6	41.3 ± 5.5	0.028
LVEF, %	60.7 ± 6.8	59.4 ± 6.9	64.1 ± 5.5	0.001
Non-PV ablation, n (%)	61 (57.5)	47 (61)	14 (48.3)	0.238
Recurrence, n (%)	36 (34)	20 (26)	16 (55.2)	0.005

BMI, Body mass index; CAD, coronary artery disease; HTN, hypertension; DM, diabetes mellitus; ACEI, angiotensin-converting enzyme inhibitors; ARB, angiotensin receptor blocker; CCB, calcium channel blocker; LAd, Left atrium diameter; LVEF, left ventricular ejection fraction

### Clinical outcomes

**Fig. 2:**
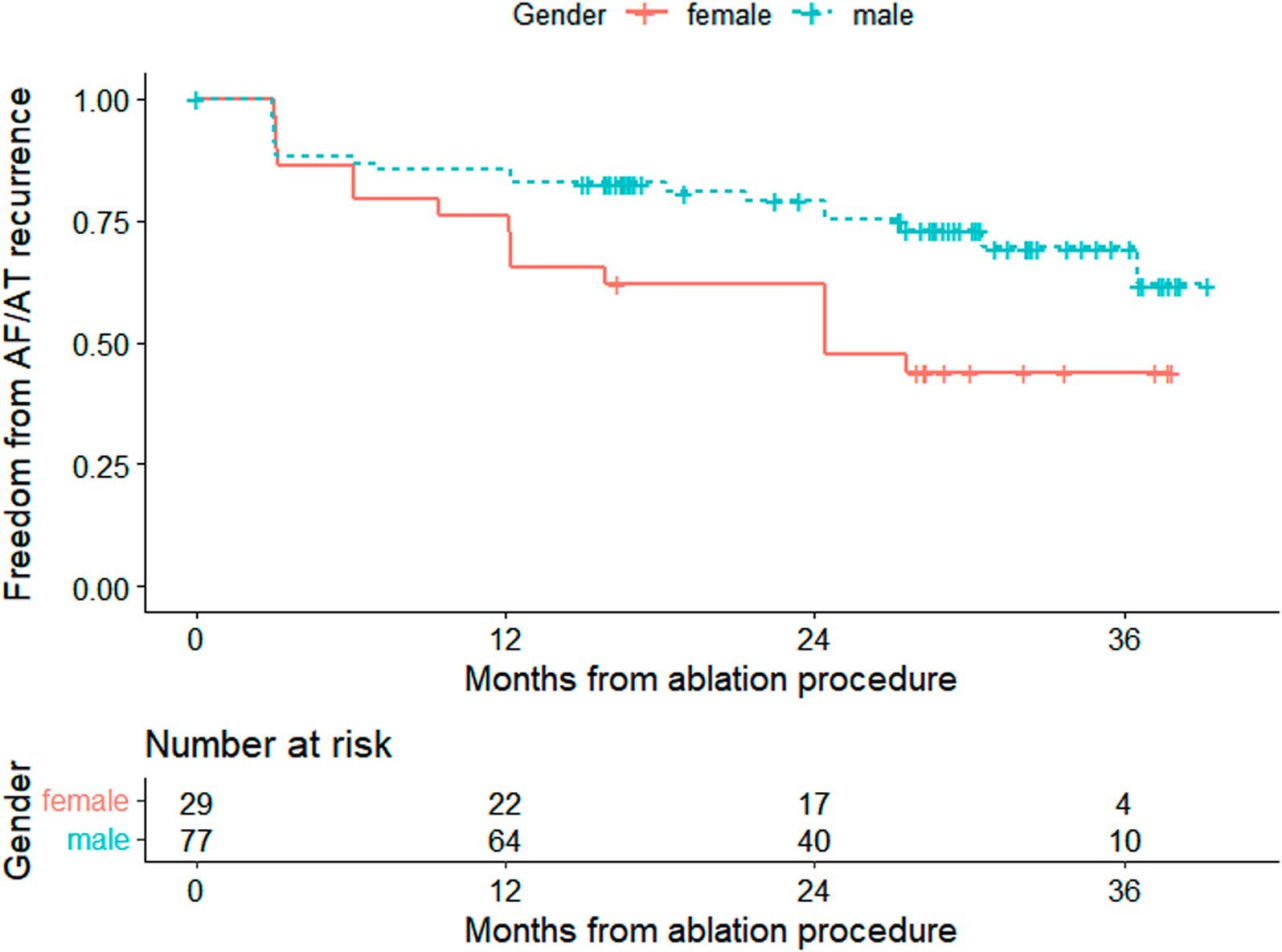
Kaplan–Meier survival curves for Freedom from AF/AT recurrence stratified by gender

The median follow-up time after RFCA was 24.4 months (interquartile range: 15.2–30.6 months), and 36 patients showed recurrence (20 men and 16 women) after AF ablation during the follow-up period. Kaplan–Meier survival analysis showed that the 12-month free from persistent AF recurrence rate was 81.7%, 86.7% for men and 72.4% for women (Additional file 1: Figure S1 and Fig. 2). Univariate Cox regression analysis revealed that non-PV ablation not associated with AF recurrence [HR: 1.003(0.516–1.947); *p* = 0.994]. Univariate Cox regression analysis showed that female sex was a risk factor for persistent AF recurrence after RFCA (Table 2) [HR: 2.099 (1.087–4.053); *p* = 0.027]. The unadjusted models revealed the relationship between sex differences and persistent AF recurrence, and the proportion of women with increasing persistent AF recurrence was unchanged after model adjustment [HR: 2.371 (1.078–5.215); *p* = 0.032, as shown in Table 3].

**Table 2:** Univariate Cox regression analysis of factors related to persistent AF recurrence

Characteristic	HR (95%CI)	*p* value
Age	1.006 (0.976–1.037)	0.695
Gender (female vs. male)	2.099 (1.087–4.053)	0.027
BMI	1.003 (0.926–1.087)	0.936
AF duration	1.006 (0.999–1.013)	0.089
*Diagnosis and treatment*		
CAD	0.719 (0.22–2.351)	0.585
HTN	1.2 (0.608–2.37)	0.599
DM	0.689 (0.243–1.952)	0.483
stroke	1.37 (0.53–3.539)	0.516
Statins	0.9 (0.41–1.975)	0.792
ACEI/ARB	1.325 (0.623–2.82)	0.465
CCB	1.096 (0.499–2.407)	0.819
Beta-block	0.939 (0.41–2.15)	0.882
EHRA score		
1	1 (ref)	
2	1.477 (0.574–3.801)	0.419
3	1.184 (0.448–3.134)	0.733
CHA2DS2-VASc score ≥ 2	1.117 (0.58–2.153)	0.741
HAS-BLED score ≥ 3	0.599 (0.082–4.381)	0.614
Non-PV ablation	1.003 (0.516–1.947)	0.994
LAd, mm (≥ 40 vs. < 40)	1.966 (0.982–3.937)	0.056
LVEF, % (≥ 50% vs. < 50%)	1.126 (0.154–8.237)	0.907

**Table 3:** Adjusted hazard ratios of post-ablation persistent AF recurrence by gender

Model adjustment		Recurrence	
		HR (95% CI)	*p* value
Unadjusted	Gender	2.099 (1.087–4.053)	0.027
Model 1	Gender	2.205 (1.114–4.364)	0.023
Model 2	Gender	2.205 (1.081–4.497)	0.03
Model 3	Gender	2.371 (1.078–5.215)	0.032

Model 1: adjusted for age (≥ 65 years, < 65 years); model 2: adjusted for age, CAD, hypertension, stroke; model 3: adjusted for age, hypertension, CAD, stroke, LVEF (≥ 50%, < 50%), BMI (≥ 24 kg/m^2^, < 24 kg/m^2^), EHRA score, CHA2DS2-VASc score (≥ 2, < 2), HAS-BLED score (≥ 3, < 3)

## Discussions

Our study revealed the following: (1) the proportion of RFCA in women was lower than that in men. (2) The risk of AF recurrence was significantly higher in women than in men. (3) Female biological sex was associated with the risk factor for AF recurrence at univariable Cox regression analysis.

Previous trails have revealed that heart failure, renal dysfunction and health status have an impact on AF recurrence after RFCA [7, 8]. To reduce the impact of comorbidities for our study, we excluded these patients in our exclusion criteria. Initially, the total numbers of persistent AF in our study are 136, and 24 patients were lost to follow-up, 6 patients with incomplete clinical information were excluded. Finally, 106 persistent AF were included in our study to analyze AF recurrence in genders after meeting the inclusion and exclusion criteria.

Sex-related differences in the epidemiology of the AF population have been reported in many studies. Women with AF are older, have a higher heart rate, have more symptoms, have a poorer quality of life and have a worse prognosis, with an increasing prevalence of stroke and death [9–11]. Similarly, our study demonstrated that female sex patients were older than male sex patients at the time of RFCA (65.1 ± 8.7 vs. 58.6 ± 10.4, respectively; *p* = 0.003), and women had higher CHA2DS2-VASc scores, which correspond to a history of stroke/transient ischemic attack.

Previous studies have disclosed that men have higher incidence rates of AF than women, and these differences may be related to sex-dependent physiological and pathophysiological mechanisms [12, 13]. Consistent with previous studies, we revealed that the LA diameter was larger in men than in women (43.7 ± 4.6 mm vs. 41.3 ± 5.5 mm, respectively; *p* = 0.028) and that LVEF was lower in men. A larger LA diameter and lower LVEF in men have been associated with an increased risk of AF and may also explain the lower incidence of AF among women.

Current studies on the outcomes of RFCA for atrial fibrillation in women are contradictory. Some studies have revealed that the AF recurrence rate was similar in women and men [14–16]. However, other studies have shown that women had lower arrhythmia-free survival following ablation than men [17, 18]. In our study, the risk of persistent atrial fibrillation recurrence after RFCA in women was significantly higher than that in men.

The exact mechanisms for sex-related differences in AF recurrence after the procedure remain incompletely understood. Among patients with AF, women may have more severe and complex arrhythmogenic substrates than men. Pak et al. reported that the LA dimension, LA voltage and pericardial fat volume were lower in women than in their male counterparts [19]. A recent study found that low voltage areas in the atrium can reflect atrial fibrotic remodeling, leading to atrial fibrillation, and the presence of low voltage zones was also a predictor for AF recurrence after catheter ablation [20]. However, Takigawa et al. revealed that the prevalence of non-pulmonary vein foci was significantly more frequent in women than in men (*p* < 0.05) [21]. Research also disclosed that compared with typical PV foci, non-PV foci are easily associated with AF recurrence after catheter ablation [22]. Therefore, non-PV foci may play a crucial role in the sex difference for AF recurrence after RFCA. However, we found that non-PV ablation not associated with gender and AF recurrence in our study. In the future, ablation of non-PV foci may be critical for maintaining sinus rhythm in women, potentially resulting in additional benefits for women and more research is needed to verify.

### Study limitations

Firstly, our study included persistent AF patients after RFCA and was a single-center study; therefore, the results may be prone to selection bias or information bias. Secondly, the AF patients included in this study were small samples; therefore, the study may have been underpowered to detect some real differences between groups. Thirdly, 24‐h Holter monitoring may lead to an underestimation of the recurrence rates compared with the implanted loop recorder. Fourthly, the results of this study may have been influenced by the inclusion and exclusion criteria, operator experience, and varying techniques used by the operators. Therefore, the generalizability of the results to other populations is unclear.

## Conclusions

In a monocentric cohort of persistent AF patients, the female biological sex was associated with a higher risk of AF recurrence after RFCA.

## Supplementary Information

**Additional file 1.** **Table S1.** Baseline clinical characteristics of patients with atrial fbrillation.

**Additional file 2.** **Fig. S1.** Kaplan-Meier survival curves for Freedom from AF/AT recurrence of all patients.

## Data Availability

The datasets used and/or analyzed during the current study are available from the corresponding author on reasonable request.

## Abbreviations

AF: Atrial fibrillation
CPVI: Circumferential pulmonary vein isolation
ECG: Electrocardiogram
HR: Hazard ratio
IQR: Interquartile range
LA: Left atrium
LVEF: Left heart ejection fraction
PV: Pulmonary vein
RFCA: Radiofrequency catheter ablation
TEE: Transesophageal echocardiography
