# Supplementary material for: Sex differences involved in persistent atrial fibrillation recurrence after radiofrequency ablation
Source: BMC Cardiovasc Disord. 2022 Dec 16;22:549. doi: 10.1186/s12872-022-03002-z (PMC9756608; doi:10.1186/s12872-022-03002-z)
Supplement: Supplementary file 1 — Additional file 1. Table S1. Baseline clinical characteristics of patients with atrial fbrillation. [file 12872_2022_3002_MOESM1_ESM.docx]

**Supplement table1 Baseline laboratory examination of the participants stratified according to gender.**

| Characteristic | Total (106) | Male(77) | Female(29) | p value |
| --- | --- | --- | --- | --- |
| RBC, 10^9^/l | 4.8±0.5 | 5.0±0.4 | 4.4±0.6 | ＜0.001 |
| WBC, 10^9^/l | 6.7±1.5 | 6.9±1.6 | 6.3±1.2 | 0.091 |
| PLT, 10^9^/l | 211.4±51.7 | 207.1±50.0 | 222.6±55.8 | 0.17 |
| Hb, g/l | 152.1±16.6 | 157.6±13.0 | 137.5±16.3 | ＜0.001 |
| eGFR, ml/min/1.73^2^ | 90.7±13.4 | 91.7±12.5 | 88.0±15.4 | 0.208 |
| GLU, mmol/l | 5.9±1.2 | 5.9±1.3 | 5.8±1.0 | 0.713 |
| UA, umol/l | 304.6±138.6 | 317.3±139.2 | 271.0±133.6 | 0.126 |
| HCY, umol/l | 16.7±9.5 | 17.6±10.0 | 14.4±7.8 | 0.115 |
| BNP, pg/ml | 157.3±122.6 | 151.2±112.0 | 174.0±148.8 | 0.42 |
| ALT, U/l | 24.6±19.9 | 26.5±22.6 | 19.4±7.6 | 0.102 |
| AST, U/l | 24.3±12.3 | 25.1±13.8 | 22.3±6.6 | 0.307 |
| LDH, U/l | 206.0±72.0 | 203.9±79.2 | 211.4±48.6 | 0.636 |
| Alb, g/l | 44.3±4.0 | 44.5±4.0 | 43.9±4.0 | 0.517 |
| Tbil, umol/l | 15.6±6.7 | 16.2±7.0 | 13.8±5.6 | 0.097 |
| Tcho, mmol/l | 4.4±1.0 | 4.4±0.9 | 4.3±1.2 | 0.583 |
| LDL-c, mmol/l | 2.6±1.0 | 2.6±1.0 | 2.4±1.0 | 0.244 |

RBC: red blood cell; WBC: white blood cell; PLT: platelet count; Hb: hemoglobin; eGFR: estimated Glomerular filtration rate; Glu: fasting blood glucose; UA: uric acid; HCY: Homocysteine; BNP: B-type natriuretic peptide; ALT: alanine aminotransferase; AST: aspartate transaminase; LDH: lactate dehydrogenase; Alb: albumin; Tbil: total bilirubin; Tcho: Total cholesterol; LDL-c: low-density lipoprotein cholesterol.

**Supplement table2. Univariate Cox regression analysis of factors related to persistent AF recurrence.**

| Characteristic |  | HR(95%CI) |  | p value |
| --- | --- | --- | --- | --- |
| RBC, 10^9^/l |  | 0.818(0.451-1.484) |  | 0.509 |
| WBC, 10^9^/l |  | 1.069(0.854-1.339) |  | 0.559 |
| PLT, 10^9^/l |  | 1.005(0.998-1.011) |  | 0.139 |
| Hb, g/l |  | 0.997(0.985-1.011) |  | 0.703 |
| eGFR, ml/min/1.73^2^ |  | 1.011(0.986-1.037) |  | 0.384 |
| GLU, mmol/l |  | 1.045(0.782-1.397) |  | 0.764 |
| UA, umol/l |  | 1(0.998-1.003) |  | 0.806 |
| HCY, umol/l |  | 0.992(0.951-1.036) |  | 0.729 |
| BNP, pg/ml |  | 1.002(0.999-1.004) |  | 0.219 |
| ALT, U/l |  | 1.008(0.997-1.019) |  | 0.152 |
| AST, U/l |  | 1.012(0.993-1.031) |  | 0.216 |
| LDH, U/l |  | 0.999(0.993-1.005) |  | 0.633 |
| Alb, g/l |  | 0.964(0.897-1.036) |  | 0.318 |
| Tbil, umol/l |  | 1.014(0.967-1.064) |  | 0.57 |
| Tcho, mmol/l |  | 1.116(0.798-1.563) |  | 0.521 |
| LDL-c, mmol/l |  | 0.768(0.523-1.128) |  | 0.179 |
