# Supplementary material for: Sex differences involved in persistent atrial fibrillation recurrence after radiofrequency ablation
Source: BMC Cardiovasc Disord. 2022 Dec 16;22:549. doi: 10.1186/s12872-022-03002-z (PMC9756608; doi:10.1186/s12872-022-03002-z)

Supplement figure.1 Kaplan-Meier survival curves for Freedom from AF/AT recurrence of all patients.


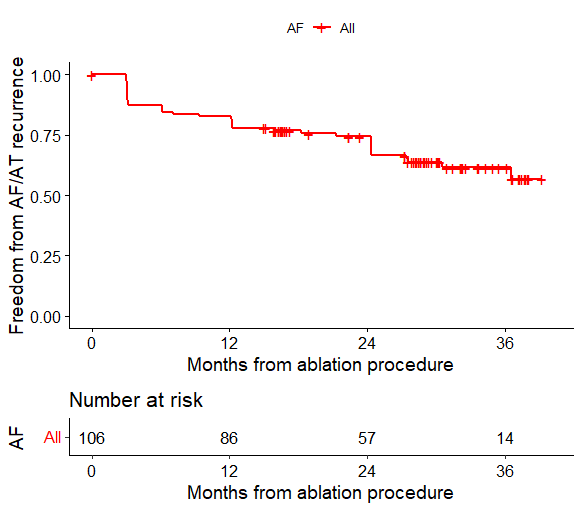

Supplement: Supplementary file 2 — Additional file 2. Fig. S1. Kaplan-Meier survival curves for Freedom from AF/AT recurrence of all patients. [file 12872_2022_3002_MOESM2_ESM.docx]
